# Perspectives on the Toxic Effects of Micro- and Nanoplastics on the Environment: A Bibliometric Analysis of the 2014 to 2023 Period

**DOI:** 10.3390/toxics12090676

**Published:** 2024-09-16

**Authors:** Xianhong Li, Zhonghong Li

**Affiliations:** 1 Hangzhou Institute of National Extremely-Weak Magnetic Field Infrastructure, Hangzhou 310028, China; 2 School of Instrumentation and Optoelectronics Engineering, Beihang University, Beijing 100191, China; 3 School of Environment and Energy Engineering, Beijing University of Civil Engineering and Architecture, Beijing 100044, China

**Keywords:** micro- and nanoplastics (MNPs), toxic effects, bibliometrics, VOSviewer, Citespace

## Abstract

Over the past decade, micro- and nanoplastics (MNPs) have garnered significant attention due to their frequent detection in and potential toxic effects on the environment and organisms, making them a serious threat to human health. To comprehensively understand the research on MNPs’ toxicity, we employed the R language-based Bibliometrix toolkit (version 4.3.0), VOSviewer (version 1.6.11) and CiteSpace (version 6.3.R1) to perform statistical and visual analyses of 3541 articles pertaining to MNPs’ toxicity between 2014 and 2023, which were retrieved from the Web of Science Core Collection (WOSCC) database. The analysis revealed that research related to MNPs’ toxicity has experienced a rapid increase in recent years. China’s particularly prominent influence in the field of MNPs’ toxicity is evidenced by its academic exchanges and the establishment of a mature cooperation system with other countries (regions), such as the USA and Germany. Studies related to MNPs’ toxicity are primarily published in leading journals, including the *Science of the Total Environment*, *Environmental Pollution*, and the *Journal of Hazardous Materials*. The Chinese Academy of Sciences was identified as the leading institution in terms of research on MNPs’ toxicity, contributing 203 papers to the total number of studies published. Keyword co-occurrence and burst analyses indicated that the current research on MNPs’ toxicity mainly focuses on the toxic effects of MNPs on aquatic organisms, the combined toxicity of MNPs and other contaminants, and the toxic effects and mechanisms of MNPs. Future research should integrate computational toxicology and toxicomics to enhance our understanding of MNPs’ toxicity mechanisms and assess the potential health risks posed by atmospheric MNPs.

## 1. Introduction

Due to their chemical stability, superior insulation properties, lightweight characteristics, and durability, plastics have substantially facilitated advancements in both industrial production and daily life. In 2017, global plastic production approached 350 million tonnes, with projections estimating an increase to approximately 1.1 billion tonnes by 2050, culminating in a staggering cumulative total of 8.3 billion tonnes over the past seventy years [1]. However, the average global recycling rate for plastics remains alarmingly low, at merely 10%, with the vast majority (approximately 90%) being disposed of through incineration, landfills, or direct release into the natural environment [2]. According to Plastics Europe, it is estimated that, annually, between 0.06 and 0.12 billion tonnes of plastic is introduced into oceans. Consequently, if the current trends continue, the cumulative amount of plastic waste in marine environments is expected to exceed 250 million tonnes by the year 2025 [3]. Furthermore, plastics do not naturally fully degrade under environmental conditions but instead decompose into particles of various sizes through a series of physicochemical and biological processes, including hydrolysis, oxidation, photodegradation, mechanical corrosion, and biodegradation. The resulting fragments from this degradation can be categorized based on their sizes into macroplastics, which are larger than 5 mm; microplastics (MPs), ranging from 1 µm to 5 mm; and nanoplastics (NPs), with dimensions of less than 1000 nm [4]. Photodegradation, induced by ultraviolet (UV) exposure, enhances the formation of oxygen-containing functional groups such as carbonyl (C=O), hydroxyl (C-OH), and ether (C-O) on plastic surfaces, thereby increasing their fragility and propensity for mechanical breakage [5]. Micro- and nanoplastics (MNPs) in aquatic environments originate from a variety of sources, including municipal drainage systems, land-based activities, fisheries, shipbuilding industries, tourism, and the daily activities of consumers and manufacturers [6]. The principal sources of MNPs in soil ecosystems include residues from agricultural activities, remnants of sludge deposits, the application of recycled water, and debris resulting from the degradation of automotive tires [7]. Furthermore, atmospheric MNPs primarily originate from fibers and paint particles released from items such as clothing and carpets [8]. MNPs can be transported through food chains, exerting effects on individual organisms, populations, and communities and ultimately impacting entire ecosystems [9,10]. These impacts accumulate and intensify over time, potentially exacerbating the degradation of fragile ecosystems [11].

The elevated concentrations of MNPs prevalent in aquatic, terrestrial, and atmospheric habitats have significant adverse implications for the wellbeing of various living organisms. MNPs can be transported through food chains, where their interconnected and amplified impacts can further degrade vulnerable aquatic and terrestrial environments [12,13]. The pervasive presence of plastics has resulted in the widespread accumulation of MNPs across diverse ecosystems. This accumulation occurs concurrently with a variety of other contaminants, including heavy metals (HMs), persistent organic pollutants (POPs), and antibiotics, compounding the environmental challenges faced by these habitats. Upon commingling, these contaminants may interact with one another, potentially altering their bioavailability and the toxicity they elicit in resident organisms. These interactions can result in synergistic outcomes, where their combined toxicity exceeds that of the individual contaminants, or antagonistic effects, where the impact is less severe than the toxicity observed from exposure to a single contaminant [14]. Over the past decade, researchers have dedicated significant efforts to exploring the toxicity of MNPs and their impact on a wide range of organisms, including fish [15], mammals [16], plankton [17], algae [18], bacteria [19], human cells [20], and plants [21]. In parallel, academic researchers have conducted comprehensive reviews of the existing literature on the toxicity of MNPs, providing a thorough analysis of the current state of knowledge in this field. For instance, Wang et al. provided a comprehensive synthesis of our current understanding of the bioavailability and toxicological implications of MPs for various fish species [22]. Ge et al. explored the physical injuries inflicted on plants by MNPs and highlighted the secondary risks associated with additives, organically derived MNP products, and the environmental contaminants adsorbed onto these particles [23]. Additionally, Anbumani et al. conducted a comprehensive review of the impact of MPs on the ecological health of biota across various trophic levels. Their analysis provided detailed insights into the processes of their uptake, accumulation, and excretion, as well as the methodologies employed for risk assessments in this field of research [24]. Although these reviews provide insights into the toxicity and adverse effects of MNPs on various test organisms, a comprehensive overview of MNPs’ toxicity is still lacking.

Bibliometrics employs mathematical and statistical methods to analyze data from the literature, thereby facilitating the visualization of co-occurrence networks and establishing a robust foundation for research in related fields [25]. Bibliometrics facilitates the summarization of the state of the scientific literature within a specific period and field, providing insights into trends in publication growth, leading authors, prominent journals, and contributing countries, thereby offering a comprehensive overview of the evolution and focal points of research across various fields [26]. Currently, bibliometrics has been extensively applied to research in various fields, such as the genotoxicity of soil organic matter [27], the toxic effects of nano-TiO_2_ [28], the toxicity of pesticides [29], and the neurotoxicity of polycyclic aromatic hydrocarbons [30], among others. To date, no reported studies have employed bibliometric methods to investigate MNPs’ toxicity.

In this study, data were derived from the Web of Science Core Collection (WOSCC) database, and bibliometric analytical tools, such as the Bibliometrix toolkit in R, VOSviewer, and CiteSpace, were utilized to facilitate a systematic analysis of the research hotspots in the field of MNPs’ toxicity from 2014 to 2023. The present study has the following aims: (1) to present a comprehensive overview of the research on MNPs’ toxicity by examining annual publication outputs and identifying primary sources; (2) to conduct an in-depth analysis of the prominent driving forces within this field, including countries, institutions, authors, and journals; and (3) to forecast emergent trends and prospective challenges associated with MNPs’ toxicity. Ultimately, this study aims to furnish readers with an unbiased and comprehensive understanding of the current state and future trajectories of the research on MNPs’ toxicity.

## 2. Materials and Methods

### 2.1. Data Sources and Search Criteria

**Figure 1:**
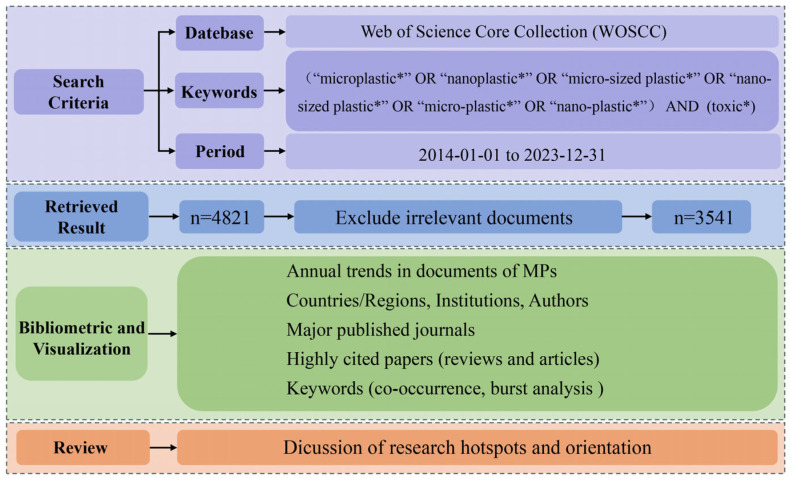
A flowchart of the bibliometric analysis and methodology used.

In the pursuit of research hotspots and trends in the field of MNPs’ toxicity, a methodological framework grounded in bibliometric analysis was adopted for this investigation. The flowchart depicting the bibliometric analysis and methodology can be viewed in Figure 1. The WOSCC database encompasses the world’s most vital and influential research literature and is acknowledged as the foremost literature search platform [31]. Our search strategy of the WOSCC database utilized the terms (“microplastic*” OR “nanoplastic*” OR “micro-sized plastic*” OR “nano-sized plastic*” OR “micro-plastic*” OR “nano-plastic*”) AND (toxic*) and covered the period from 1 January 2014 to 31 December 2023. The literature was further screened based on titles and abstracts, and articles not relevant to the study of MNPs’ toxicity were excluded. Upon screening the search results, any conference abstracts, editorial materials, book chapters, and other materials with limited relevance to the topic were eliminated. The resulting collection of articles (n = 3541) related to MNPs’ toxicity was subjected to a comprehensive research evaluation and scientific mapping. The dataset comprised 2945 research articles and 596 review papers. The data exported from the WOSCC database were exported using the “fully documented and cited references” in TXT format, with the literature search ending on 25 March 2024.

### 2.2. Analysis Method

A bibliometric analysis is a quantitative statistical methodology applied to the literature within a specific research field to elucidate research hotspots and development trends [32]. Bibliometrix (version 4.3.0) is an R-based bibliometric package designed for information analyses of the literature and visual map creation that enables the comprehensive examination of a particular field’s research and the identification of its hotspots from multiple perspectives [33]. VOSviewer (version 1.6.11) is a bibliometric visualization software tool designed to enable the generation of intricate network maps, thereby providing an accessible means of mapping the complex relationships in academic research [34]. Additionally, CiteSpace (version 6.3.R1) is a visualization tool developed by Dr. Chaomei Chen to evaluate and analyze data from the literature. Its burst term analysis function detects terms exhibiting large rates of change in their use over a specified period from within a vast collection of subject words via keyword frequency analysis and is used to understand the frontiers of research in an area [35].

In this study, articles downloaded from the WOSCC database in TXT format were imported into the Bibliometrix toolkit, VOSviewer, and Citespace software. Keywords with identical or similar meanings were unified and consolidated into one keyword. For instance, the keywords “microplastic” and “microplastics” were unified to “microplastic”. VOSviewer software was employed to analyze the data related to published papers, collaborative relationships, keywords, and other information in the field of MNPs’ toxicity, leading to the construction of a visual knowledge graph. The keyword burst analysis function of Citespace was used to understand the evolution of research related to MNPs’ toxicity. SCImago Graphica (version 1.0.42) was utilized to generate relevant network diagrams of the collaborative relationships between countries. Data compilation was executed with the assistance of the Bibliometrix toolkit software in R, while the related figures were created using Origin (version 2018).

During the preparation of this manuscript, the authors employed ChatGPT 4.0 to conduct spelling checks and linguistic polishing. Subsequent to utilizing this tool/service, the authors meticulously reviewed and revised the content as necessary.

## 3. Results and Discussion

### 3.1. Publication Outputs and Subject Categories

**Figure 2:**
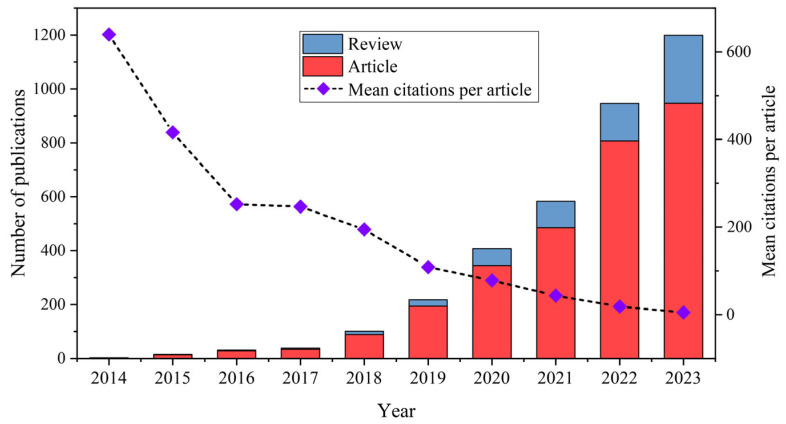
Number of publications and mean citations per article on MNPs’ toxicity during the 2014–2023 period.

The temporal distribution map of publications associated with this research field grants us valuable insights into the degree of attention they garnered and the progression of trends in the field, thereby elucidating its development over time [36]. Figure 2 illustrates the growth trend in publications related to MNPs’ toxicity during the period analyzed (2014–2023). The majority of publications from the past decade, specifically over 75%, were published in the past three years. As depicted in Figure 2, a comprehensive assessment of the literature from 2014 to 2023 related to MNPs’ toxicity revealed a continual upward trajectory. The number of published articles rose from a meager 3 in 2014 to a substantial 1199 in 2023. Furthermore, between 2020 and 2023, the rate of articles published annually remained steady, at approximately 266. Notably, the year 2022 witnessed a remarkable surge in the number of articles published, which increased by 363 compared to the previous year, thereby exceeding the average growth rate observed. Between 2014 and 2023, the cumulative citations of articles in this field reached 149,024, and the mean citations per article reached 42.08. Despite the relatively modest volume of publications in the initial years of this study, these early papers were citated more frequently, averaging over 200 citations per paper until 2017. This trend underscores the significant impact of and attention garnered by the foundational research published during this period. However, as the volume of publications expanded in subsequent years, there was a gradual decline in the average citation rate of these articles annually, reflecting the dilutive effect of the increasing number of papers.

**Figure 3:**
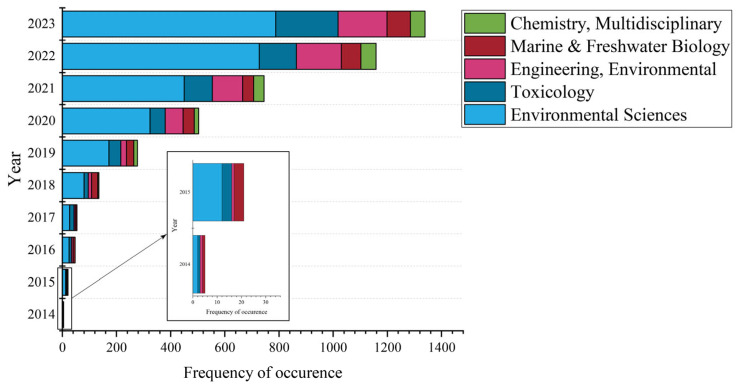
Top 5 subject categories related to research on MNPs’ toxicity, published from 2014 to 2023 in the WOSCC database.

According to the WOSCC’s discipline categories, a total of 89 disciplines were engaged in the study of MNPs’ toxicity, with the top 5 represented in Figure 3. Research pertaining to MNPs’ toxicity was predominantly concentrated within the realm of environmental sciences, followed closely by engineering, environmental studies, and toxicology. Together, these fields account for over 65% of the total number of articles published. Certain publications demonstrate a merging of multiple disciplines, thereby necessitating the classification of individual articles into several distinct, yet interconnected, categories. Research on MNPs’ toxicity extends beyond environmental sciences and toxicology to chemistry; multidisciplinary studies; marine and freshwater biology; and public, environmental, and occupational health, revealing its cross-disciplinarity and the interplay seen among various fields. Furthermore, the fundamental disciplines in this area—environmental sciences, toxicology, chemistry, multidisciplinary studies, and marine and freshwater biology—have expanded across a broader array of scientific disciplines, exhibiting considerable potential for development.

### 3.2. Relevant Countries/Regions

The number of publications originating from a country can indicate the extent of research activity in a particular field [37]. Collectively, the top 10 countries publishing studies on MNPs’ toxicity produced 76.84% of all published articles, thus representing a driving force in this research. As illustrated in Table 1, China assumes a vanguard position, contributing an impressive 1570 articles to the total published articles and markedly outperforming other countries. Next in line are Italy and South Korea, with 195 and 168 articles, respectively. Within the top 10 contributing countries, the articles exclusively authored by domestic researchers surpass those featuring multinational authorship, indicating that the degree of international collaboration in this research field warrants further expansion and development. In terms of overall citation frequency, China, Italy, and the USA occupy the top three positions, with China far in the lead, amassing approximately 5.44 times the number of citations Italy had, which ranked second. Regarding the average citation frequency, Portugal, the USA, and Germany hold the top three spots, with 76.13, 60.32, and 57.98 citations per paper, respectively. This demonstrates that the scientific research results generated by these three countries have a considerable influence on the field and are widely read and quoted.

**Figure 4:**
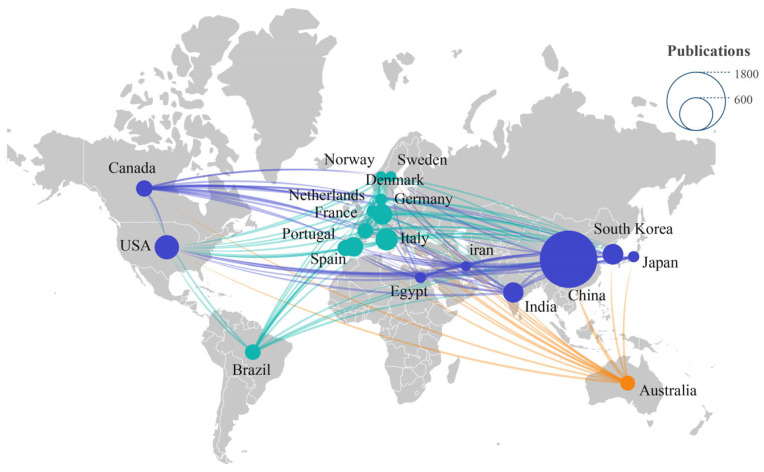
The top 20 countries/regions in terms of the number of publications generated that relate to MNPs’ toxicity between 2014 and 2023.

Based on the statistical results of the network map visualization analysis, the collaborative relationships between countries that are related to research published on MNPs’ toxicity from 2014 to 2023 are depicted in Figure 4. As observed in Figure 4, various countries/regions collaborate in order to investigate the toxicity of MNPs, with the USA, Canada, South Korea, Japan, and India being the main countries cooperating with China.

### 3.3. Relevant Institutions and Authors

Publishing institutions represent the primary contributors to scientific research. Through the statistics of these institutions, the overall research input and its influence in a specific field can be more vividly displayed [38]. From 2014 to 2023, a total of 3199 research institutions studied MNPs’ toxicity. Table 2 reveals the institutions with the largest numbers of articles published in this field, with the Chinese Academy of Sciences at the top of the list (104 articles, 5.73%). Following closely are the University of Chinese Academy of Sciences (103 articles, 2.91%) and Nanjing University (76 articles, 2.15%) in the second and third positions, respectively. A total of six research institutions had over 60 publications each, with the Chinese Academy of Sciences standing out, as it remarkably surpassed the output of the other institutions. When examining their geographical distribution, nine of the top ten research institutions are based in China, and one is located in Portugal. This underscores China’s preeminence in this research field.

Evaluating the number of articles published by an author and their corresponding citation counts enables the discernment of highly prolific and impactful researchers in a specific field, thereby highlighting their contributions to its advancement [39]. In the field of MNPs’ toxicity, 12,018 authors contributed to the existing body of literature. The most prolific contributors are Wang J (79 articles), Wang DY (54 articles), and Wang Y (50 articles) (Table 3). Notably, the majority of the articles by the top 10 authors were published between 2020 and 2023, indicating a burgeoning interest in the field of MNPs’ toxicity and a concomitant upsurge in research during this period. For instance, the European Commission formally introduced MP pollution as a topic for the European Plastics Strategy in January 2018. In its Zero Pollution Action Plan, released in May 2021, the European Commission stated its aim to reduce MP emissions by 30% by 2030. Furthermore, the heightened awareness of emerging contaminants (ECs) in recent years has also spurred the rapid advancement of research on MNPs’ toxicity.

### 3.4. Relevant Journals

Influential journals in a research field can be identified through the analysis of sources from the literature [40]. Through the analysis of the 3541 sources from the literature included in the analysis, the results indicate that 415 journals contributed to this field. The top ten journals are delineated in Table 4, with the foremost three being *Science of The Total Environment*, *Environmental Pollution*, and the *Journal of Hazardous Materials*, boasting 515, 323, and 309 articles each. It is worth highlighting the observation that *Environmental Science & Technology*, while contributing 87 articles to the field, boasts an exceptional average citation score of 119.86, underscoring the considerable impact of its published works. This journal has a substantial influence on its counterparts, as it is the only journal in the top 10 with an average citation score that surpasses 100. The *Journal of Hazardous Materials* has had the highest average impact factor over the past five years, which was 13.6. *Environmental Science & Technology* and *Science of The Total Environment* occupied the second and third positions, respectively, in terms of their influence within the field. According to the Journal Citation Reports (JCR) classification standards, articles on MNPs’ toxicity were predominantly found in environmental and toxicology journals. These top 10 journals all qualified as Q1 journals in the JCR, publishing a comparatively greater quantity of high-level research on MNPs’ toxicity and exerting a significant influence on this field.

### 3.5. Highly Cited Articles and Reviews

Highly cited studies in the literature in a field is not only the core body of work that is widely acknowledged and frequently referenced in that field, but also serves as crucial foundational knowledge and a methodological basis for future research [41]. Table 5 presents the top 10 most cited publications pertaining to MNPs’ toxicity. In terms of their research, the primary focus of these articles is predominantly the examination of the toxic effects exerted by MNPs on various representative model organisms (zebrafish, *Arabidopsis thaliana*, mice, *Caenorcaditis elegans*, etc.) and aquatic organisms (marine mussels and *Brachionus koreanus*).

In the realm of research on MNPs’ toxicity, the most frequently cited publication (article) from 2014 to 2023 was an article titled “Uptake and accumulation of polystyrene microplastics in zebrafish (*Danio rerio*) and toxic effects in liver”, which was authored by Lu YF’s team and published in *Environmental Science & Technology* in 2016, with a total citation frequency of 1210. Another notable publication was that by Lei L’s team, titled “Microplastic particles cause intestinal damage and other adverse effects in zebrafish *Danio rerio* and nematode Caenorhabditis elegans”, which came third, with 788 citations. Zebrafish is a small freshwater fish measuring approximately 2–3 cm, characterized by its diminutive size, brief life cycle, and robust reproductive capacity. It shares 70% gene homology with humans and has an overall genome similarity of 87%, with 82% of human disease-related genes being identifiable in zebrafish. Consequently, zebrafish are often employed as model organisms for assessing the toxicity of MNPs, which is carried out by examining their bioaccumulation and toxic effects on the organism’s development, reproduction, cardiovascular system, neurodevelopment, and ocular health [42]. Lu et al. focused on scrutinizing the absorption and subsequent tissue accumulation of polystyrene microplastics (PS-MPs) in zebrafish while also delving into the deleterious ramifications of these pollutants on their liver function [43]. Meanwhile, Lei et al. evaluated the effects of five prevalent MPs (polyamide (PA), polyethylene (PE), polypropylene (PP), polyvinyl chloride (PVC), and polystyrene (PS) particles) on zebrafish. Their findings revealed that exposure to MP primarily led to intestinal damage and oxidative stress in the studied organisms [44]. The second most frequently cited article was that by Avio C G’s team, titled “Pollutants and toxicological risk from microplastics to marine mussels”, which was cited 854 times. Their study discovered that PE and PS MPs could adsorb pyrene and other organic pollutants while bioaccumulating in marine mussels. Consequently, this exposure instigated a cascade of physiological changes in marine mussels, including alterations in their lysosomal compartment, immunological responses, antioxidant system, peroxisomal proliferation, neurotoxic effects, and the induction of genotoxicity [45].

Table 6 lists the 10 most cited publications (reviews) related to MNPs’ toxicity. In terms of their content, the primary emphasis centered on elucidating the toxic effects of MNPs on human health, as well as their ramifications for aquatic organisms. Two of these review articles were authored by Prata J C’s team, titled “Environmental exposure to microplastics: An overview on possible human health effects” [46] and “Airborne microplastics: Consequences to human health?” [47], and are the first and second most cited papers, respectively. The reviews delved into the exposure pathways (including ingestion, inhalation, and dermal contact) and various toxic effects (including oxidative stress, inflammatory lesions, and augmented intake or translocation) of MNPs on the human body. Additionally, they scrutinized the potential risks associated with airborne MNPs and their ramifications for human health. The third most cited review, by Bouwmeester H’s team, is titled “Potential health impact of environmentally released micro- and nanoplastics in the human food production chain: experiences from nanotoxicology” and was published in *Environmental Science & Technology* in 2015. This review systematically assessed the potential health risks associated with the presence of environmentally released MPs in the human food production chain. This investigation encompassed not only the MPs themselves, but also the POPs adsorbed onto MPs and the leaching of plastic additives from these particles [48].

### 3.6. Keyword Analysis

#### 3.6.1. Keyword Co-Occurrence and Thematic Evolution Analysis

**Figure 5:**
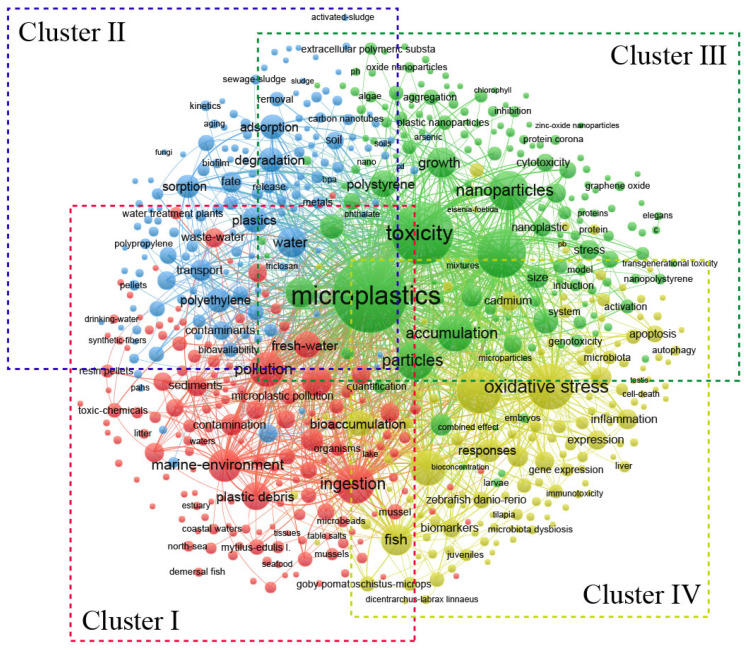
Network visualization of keyword co-occurrence network.

Keywords provide a highly condensed representation of an article’s content. The statistical analysis of keywords allows for an intuitive understanding of an article’s central idea and theme and can reflect research hotspots and development trends in the field [49]. A keyword co-occurrence analysis involves calculating the probability of the occurrence of a set of words within the same field and, based on this probability, classifying significant keywords or subject words in that research area according to the Euclidean distance between the words, thereby revealing the field’s hotspots, structure, and paradigms [50]. The VOSviewer software was employed for a co-occurrence analysis of keywords, resulting in a cluster map (Figure 5). In this map, the nodes represent keywords, and their size indicates the frequency of their occurrence, with larger nodes corresponding to higher frequencies. The lines between nodes represent keyword co-occurrence, with line thickness denoting the strength of the connection. Identically colored nodes belong to the same cluster. These clustering results reveal that the research field’s content mainly encompasses four topic clusters (Table 7).

Cluster I (the red cluster): The main keywords in this cluster are “marine environment”, “ingestion”, “acute toxicity”, “*Daphnia magna*”, and “zooplankton”, suggesting that the primary research focus in this cluster was the impact of MNPs on aquatic organisms. Compared to terrestrial ecosystems, polymers break down and degrade more readily into smaller plastic fragments in aquatic ecosystems, making them a significant source of MNPs [51]. MNPs, and the chemical pollutants they harbor, have the capacity to influence the growth and reproduction of aquatic organisms. Through the mechanisms of the food chain and trophic web, these materials may be transmitted to higher organisms, eventually accumulating within humans and subsequently posing potential threats to human health [46]. Therefore, studying the toxic effects of MNPs on aquatic organisms is crucial. Common aquatic ecosystem groups include bacteria, actinomycetes, protozoa, microalgae, plankton, and fish. Once MNPs enter the aquatic environment, they readily interact with smaller aquatic organisms, including microalgae.

Smaller aquatic organisms and their larvae constitute a crucial nutritional bridge between primary producers and higher-level aquatic organisms. The adverse effects of MNPs on the growth, development, and behavior of these diminutive organisms could exert a substantial influence on their population size and structure. Consequently, these impacts have the potential to disrupt the normal functioning of aquatic ecosystems. Through biological transfer, microalgae carrying MNPs can be consumed by higher-level organisms (such as primary consumers). The transfer between trophic levels may result in a large number of MNPs entering the aquatic food web and being assimilated by aquatic biota, causing harm to the system’s balance and the normal metabolism of organisms [52]. The distribution of MNPs across various trophic levels and their accumulation within organisms may significantly impact their propagation through the food chain or food web, consequently posing a grave risk to human health. The identification of MNPs has occurred within aquatic organisms from higher trophic levels. In a pioneering study, Murray and Cowie illustrated the transfer of MNPs from prey to predator along the food chain, which they achieved by providing lobsters with fish containing PP fibers [53]. This phenomenon showcases the biological transfer of MNPs, which is accompanied by their escalatory accumulation within these higher levels. Despite these findings, there remains a paucity of research and documentation explicitly addressing the transfer of MNPs to humans and any subsequent ramifications on human health.

The possibility of the transmission and amplification of MNPs in the food chain is inextricably linked to marine life and, ultimately, human populations. Presently, the majority of studies investigating the toxic effects of MNPs on aquatic organisms primarily address singular impacts, with less consideration given to broader food chain implications. Future research endeavors should augment investigations into the toxic accumulation and transformation of MNPs in the food chain, explore the transfer effect of MNPs within food chain dynamics, and create a foundation for the prevention and mitigation of MNPs’ impact on ecological environments and human health. Moreover, forthcoming studies ought to encompass entire ecosystems, examining the interdependencies between biotic populations and communities, with a particular emphasis on food chain mechanisms, material cycling, and energy flow processes. This comprehensive approach will facilitate the assessment of the global ramifications of MNP pollution on ecosystems and uncover the long-term consequences of MNP contamination on biotic populations and communities.

Cluster II (blue cluster): The main keywords in this cluster are “polycyclic aromatic hydrocarbons”, “heavy metals”, “bisphenol A”, and “flame retardants”, indicating that the combined toxicity of MNPs and other contaminants was the primary research focus in this cluster.

Environmental media characteristically contain a number of contaminants rather than just one, meaning that researchers must account for the combined toxic effects of multiple contaminants on organisms when evaluating their ecotoxicity. MNPs exemplify this complexity as they possess an expansive specific surface area and pronounced hydrophobicity. These properties enable MNPs to adsorb various contaminants, including HMs, pharmaceuticals, personal care products (PPCPs), endocrine disruptors (EDs), and antibiotic resistance genes (ARGs), from diverse environmental media [54]. When these contaminants are mixed together, interactions occur that alter their bioavailability and toxicity to organisms. The interplay between these contaminants may culminate either in a synergistic effect, wherein the combined toxicity of the pollutants surpasses that of an individual contaminant, or an antagonistic effect, characterized by a reduction in overall toxicity when multiple pollutants interact. Some studies have shown that the combined toxicity of these pollutants is not simply synergistic or antagonistic and that the toxic effects and antagonistic mechanisms experienced by organisms are not as simple as “1 + 1”. Traditional risk assessments are based on single exposures, which might underestimate or overestimate the potential risk to organisms. For example, Qiao et al. discovered that the presence of natural organic matter facilitated the absorption of copper by PS MPs with dimensions of 0.1 μm and 20 μm [55]. In contrast, Fu et al. found that the toxic effects exerted by PVC MPs and copper on microalgae changed after they were aged by seawater [56]. These differing results may depend on MNPs’ capability to adsorb HMs. A strong affinity between pollutants can lead to antagonistic effects, while a weak affinity demonstrates synergistic effects. Considering the intricate interplay between MNPs and HMs, coupled with their effects on aquatic biota, there is an urgent need for expanded research in this field. Presently, the majority of MNP research is focused on primary MNPs, with a limited number of studies exploring the toxic effects of secondary MNPs, which are created through environmental degradation processes. Secondary MNPs, due to their greater specific surface area, are more predisposed to adsorbing contaminants and releasing chemicals embedded within MNPs. This, in turn, could potentially augment the toxicity associated with these MNPs [57].

Researchers must study the indirect toxic effects of MNPs in addition to their direct toxic effects. During the fabrication of plastic products, a wide variety of additives, including antioxidants, antistatic agents, blowing agents, flame retardants, lubricants, impact modifiers, plasticizers, colorants, and filler materials, are used to enhance their processing performance, improve efficiency, and reduce production costs [58]. These additives may include EDs and other harmful substances. The majority of additives incorporated into plastics lack covalent bonding, which renders them susceptible to migration towards the surfaces of MNPs when fragmented or degraded due to biotic or abiotic factors. Once liberated, certain additives, such as bisphenol A, may present hazards to human health that exceed those directly attributable to MNPs alone [59]. MNPs and their released additives can harm the endocrine system and organs, increase the risk of various health problems, and even affect reproductive development and fertility [60]. Presently, the data from experiments on MNPs’ toxicity likely relate to the additives within MNPs rather than MNPs’ inherent toxicity. MNPs can function as carriers of additives that accelerate their migration into organisms and eventually transfer them to higher-level organisms.

Cluster III (green cluster): The main keywords in this cluster are “growth”, “accumulation”, “health”, “behavior”, and “size”, indicating that the primary research focus in this cluster was the toxic effects of MNPs. Due to differences in species, living habits, and trophic levels among microorganisms, algae, plants, fish, and mammals, the toxicological effects of MNPs on various organisms can differ significantly [61]. Contemporary research examining the toxic effects of MNPs encompasses investigations into species’ biological survival, growth, reproduction, feeding activity, physiological and biochemical parameters, histopathology, and gene expression levels. The ecotoxic effects of MNPs are multifaceted, resulting in a wide array of impacts on biological growth and function. Nonetheless, their precise effects may be contingent on factors such as the plastic’s polymer type, bioavailability, and particle size; the exposure dose and its duration; the biological species affected; and the experimental conditions [62]. The physical and chemical attributes of MNPs are intimately connected to their bioavailability and toxicity within ecological contexts. For instance, the toxic effects exhibited by MPs exhibit a marked size dependence, whereby smaller particles demonstrate an increased likelihood of uptake by organisms and subsequent accumulation in their systems. Overwhelmingly, greater toxicity tends to be induced by NPs [63], as when ingested by organisms, smaller MPs may release more toxic substances, exacerbating their toxic effects. The size of the MPs also influences the behavior of organisms towards them. For instance, in fish and zooplankton, MPs that resemble food in size and color may be ingested unintentionally and affect their feeding behavior by interfering with food selection. Smaller MPs might be overlooked or even mistaken for digestible nutrients [64]. Due to the inherent complexity and diversity of MNPs, as well as the vast array of their sources, additional research is warranted to elucidate the influence of MNPs’ varying physical and chemical properties on organisms in environmental settings and the subsequent emergence of toxic effects.

The majority of current studies rely on laboratory settings and high-dose, short-term exposure models. Future research should focus on low-dose, long-term exposure models that more closely resemble actual environmental conditions and avoid overemphasizing the toxicological effects of MNPs. In addition, most current studies concentrate on common MNPs, such as PE, PP, and PVC. Upcoming research should examine a broader spectrum of MNP materials, including biodegradable plastics, polyamides, and polyether alcohols, to reveal the differences in biochemical toxicity among the various MNPs affecting organisms. Moreover, future research should develop more scientific, unified, and reliable assessment methods and standards, establish toxicological indicators, and construct a more comprehensive evaluation and detection system for toxic effects. This would enable the accurate assessment and monitoring of MNPs’ biochemical toxicity on a global scale.

Cluster IV (yellow cluster): The main keywords in this cluster are “oxidative stress”, “bioaccumulation”, “biomarkers”, “expression”, “responses”, and “DNA damage”, indicating that the primary research focus in this cluster was the toxicity mechanisms of MNPs. These mechanisms can be broadly divided into two categories: mechanical and physical damage and biochemical toxic damage [65]. For bacteria, the mechanical and physical damage caused by MNPs is evident in terms of cell wall destruction, which impacts bacterial attachment and aggregation. This interference can affect the formation of biofilms by soil or aquatic bacteria, consequently influencing the stability and ecological function of the ecosystems they inhabit [66]. For algae, damage occurs through shading effects, physical adsorption, and physical damage to their cell walls and membranes [67]. In fish, the ingestion of MNPs can cause digestive tract damage, osmotic pressure imbalance, internal organ damage, and behavioral abnormalities [68]. For plants, MNPs can cause physical damage to roots by blocking the space between the cell wall and seed coat surfaces, thus affecting water and nutrient absorption, as well as photosynthesis [69]. In mammals, damage affects the digestive tract, respiratory tract, internal organs, and reproductive system [70].

At the bacterial level, MNPs’ biochemical toxic damage to organisms triggers oxidative stress responses and inhibits growth, reproduction, and vitality. NPs can even cause lethal damage to certain bacteria (e.g., *Streptomyces cerubicans*) [71]. In plankton, MNPs lead to oxidative stress, metabolic disorders, growth inhibition, and reduced reproductive capacity in offspring. They can also disrupt the balance of reactive oxygen species, causing lipid oxidation and inhibiting algal growth [72]. In plants, the absorption of MNPs triggers oxidative stress, leading to oxidative damage in plant proteins, lipids, and nucleic acids, which, in turn, affects plant growth and induces cytotoxic and genotoxic effects [73]. In fish, the biochemical toxicity of MNPs causes behavioral disorders, metabolic abnormalities, growth retardation, DNA and gene expression alterations, cell apoptosis, oxidative stress, inflammatory responses, and neurotoxicity [74]. In mammals, the biochemical toxicity mechanisms of MNPs include endocrine disruption, oxidative stress, cytotoxicity, immune system damage, and neurotoxicity, potentially impairing growth, development, reproduction, and other physiological functions [75].

The current research on MNPs’ toxicological mechanisms remains limited, and the specific biochemical toxicity mechanisms of MNPs are yet to be thoroughly explored. Much of the existing research focuses on inflammation and oxidative stress, with less attention being paid to deeper molecular-level mechanisms. Future studies should employ advanced methodologies to enable more in-depth experimental investigations, particularly those examining the processes occurring at the molecular level, such as the activation of signaling pathways, changes in gene expression, and metabolic abnormalities.

#### 3.6.2. Keyword Burst Detection Analysis

**Figure 6:**
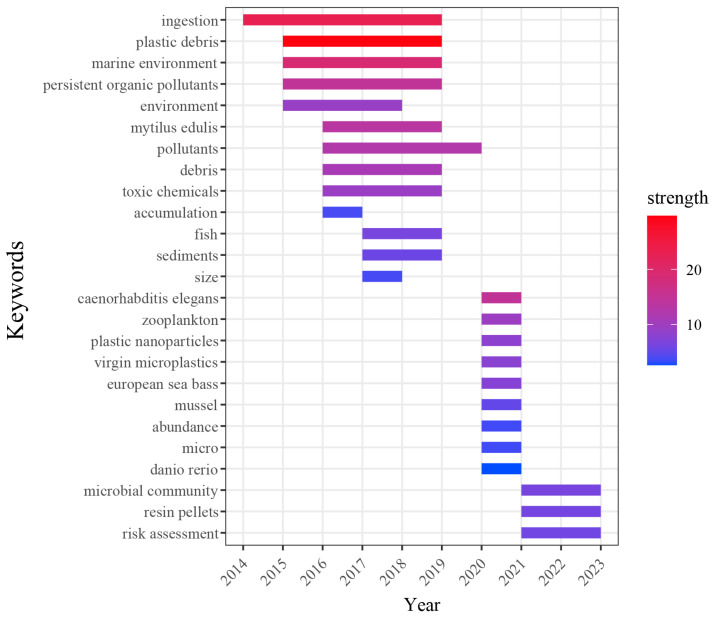
The top 25 keywords with the strongest citation burst in articles on MNPs’ toxicity published from 2014 to 2023.

To gain an understanding of the most recent advancements in the research on MNPs’ toxicity, it is imperative to conduct a dynamic analysis of the frontier of this research, which shifts over time and embodies the focal points of the field. A research frontier can be discerned and monitored by extracting and examining emerging subject keywords. By employing CiteSpace software’s burst detection capabilities, a network map depicting the top 25 keywords with the highest citation burst strength was generated (Figure 6).

During the nascent exploration stage of MNPs’ toxicity (2014–2019), the prevailing keyword was “marine environment”, suggesting that initial research efforts in this area were concentrated on the toxic effects of MNPs on aquatic organisms inhabiting marine ecosystems. MNPs pervade the global marine environment, with an estimated annual influx of 11 million tons of plastic waste. Approximately 5.12 × 10^13^ plastic debris fragments are dispersed in the surface layer of the world’s marine environments, and projections for 2050 indicate that the volume of plastic waste in oceans may surpass that of the fish population [76]. MNPs exert varying degrees of influence on the constituents of marine ecosystems, including producers (e.g., phytoplankton and algae), consumers (e.g., fish, crustaceans, and shellfish), and decomposers (e.g., bacteria and fungi). Their toxic effects manifest in these organisms’ growth, reproduction, and survival functions and potentially disrupt the balance of the marine ecosystem through indirect means such as mutation, competition, and infection. Keywords like “persistent organic pollutants” and “toxic chemicals” also had a high burst strength, reflecting the relationship between MNPs and POPs in two ways: First, MNPs’ excellent surface adsorption ability enables them to adsorb various POPs (e.g., polycyclic aromatic hydrocarbons, polychlorinated biphenyls, and DDT) and act as transport carriers for chemical pollutants in marine ecosystems. Second, manufacturers often add organic additives (plasticizers, flame retardants, and antioxidants) during plastic product production, which may be released during MNPs’ decomposition due to changes in their molecular structure and chemical stability [77]. Consequently, marine organisms might ingest MNP particles rich in organic pollutants, which may be released internally, causing adverse biological effects (e.g., endocrine disorders, changes in biological enzyme activities, and immune impairment). Moreover, the enrichment effects of POPs in biological tissues may intensify as they are transmitted through the food chain.

In recent years (2020–2023), the emergence and intensity of keywords such as “*Caenorhabditis elegans*” and “*Danio rerio*” have increased. These organisms are considered typical model organisms for studying MNPs’ toxicity as they can reveal the biological effects, physiological changes, and potential ecological risks that result from exposure to MNPs. Additionally, the intensity of the emergence of the keyword “microbial community” was high during this period. Within an ecosystem, microbial communities are responsible for sustaining critical biochemical cycles, including the decomposition of organic matter, nitrogen fixation, and nutrient cycling. The toxic effects of MNPs on microbial communities may directly or indirectly influence their structure and diversity, impacting their functional gene expression and leading to dysfunction in the organic matter decomposition and nutrient cycling processes. These changes ultimately affect the ecosystem’s operation and biochemical cycling [78]. As microbiology and genomics technology continue to develop, research on the population structure, metabolic pathways, and gene expression of microorganisms affected by MNPs will deepen, facilitating a better understanding of the environmental and ecosystem impacts of MNPs and promoting the prediction and prevention of potential risks.

## 4. Avenues for Future Research

### 4.1. The Application of Computational Toxicology to Evaluate the Toxicity of MNPs’ Additives

The wide variety, diverse functions, and large numbers of plastic additives used in plastics render the evaluation of their environmental impact highly challenging. Current methodologies for testing the toxicity of plastic additives include traditional in vivo experiments, high-throughput in vitro experiments, and computational toxicology methods leveraging computer simulation predictions or in silico techniques. Traditional animal models employed for evaluating chemical toxicity are frequently characterized by high costs and time-intensive processes and often prove inadequate in detecting substances detrimental to human health due to interspecies variability. While in vitro experiments curtail the necessity for animal usage, their testing environments might not sufficiently emulate the intricacies of natural ecosystems and the multifarious interactions that transpire among organisms [79].

Rapid advancements in structural biology and machine learning have substantially increased the application of computational toxicology methods within environmental toxicology. Computational toxicology, an emergent discipline that combines computer science, chemistry, and biology, strives to predict the toxicity and environmental impacts of chemicals through computational models and algorithms. Such methods can predict the toxicity of chemicals adsorbed on MNPs’ surfaces by constructing quantitative structure–activity relationship (QSAR) models. Moreover, methodologies like quantum mechanics/molecular mechanics (QM/MM) and physiologically based pharmacokinetic (PBPK) approaches can elucidate the specific molecular mechanisms underlying the toxic effects of MNPs on organisms and help develop environmental risk assessment models to evaluate their impacts on ecosystems.

Computational toxicology methods are cost-effective and rapid and do not require experimental animals. The adverse outcome pathway (AOP) links the adverse outcome (AO) of a chemical at the individual level to its molecular initiating event (MIE) at the molecular level. Given that AOPs are structured across molecular/cell/organ/individual/population levels, toxicity events occurring at higher levels, such as organ, individual, or population levels, may be inferred from lower-level toxicity events, such as those transpiring at the molecular or cellular levels [80]. These results facilitate the application of in vitro biological assay data to the high-throughput toxicity prediction of chemicals. Jeong et al. devised an AOP grounded in biological activity classification mechanisms for 50 prevalent additives and a toxicological correlation analysis in mammals. Their findings indicated that the principal toxicity pathways associated with these additives include oxidative stress, carcinogenesis, inflammation, and disruptions to lipid metabolism [81]. Future toxicity studies of plastic additives will likely embrace a more high-throughput approach. However, due to the limitations of these models and prediction methods, computational toxicology may not accurately predict the toxic effects of specific additives.

### 4.2. The Application of Omics Techniques to Assess the Toxicity of MNPs

The rapid advancements in bioinformatics and high-throughput sequencing technologies have prompted researchers to shift from traditional toxicology testing methods to faster, more sensitive, and more efficient approaches. Omics technology, which encompasses genomics, proteomics, and metabolomics, has significantly advanced our understanding of biology and medicine and has become widely adopted in MNP toxicology studies [82]. The progress in genomics has enabled researchers to overcome the limitations of previous environmental toxicology experiments, which focused solely on biological structure, function, and behavior indicators, by employing high-throughput methods to detect molecular changes (genes, proteins, and metabolites) following an organism’s exposure to external stressors and thus obtaining microbial genomic information. Moreover, genomics provides transcription annotations, function predictions, and other information that can elevate toxicology research, improving upon the limitations and relative shortcomings of traditional toxicology. This approach allows for the examination of MNPs’ toxicity mechanisms on test organisms at biochemical, genetic, cellular, protein, and other molecular biological levels [83]. In the future, traditional biological experimental methods could be combined with bioinformatics techniques, such as proteomics, transcriptomics, and metabolomics, to investigate the interactions between MNPs’ effect on genes, proteins, and enzymes. By analyzing gene expression, the metabolic states of organisms can be assessed to further explore MNPs’ pathogenic mechanisms of action on organisms and their toxic effects.

### 4.3. Atmospheric MNPs and Toxicity

Currently, most research on MNPs investigates their occurrence, migration, and transformation in freshwater, soil, and ocean environments or the toxicity of MNPs to aquatic and soil organisms. The atmosphere, a significant exposure route and environment for MNPs, has often been overlooked. However, recent years have witnessed growing attention being paid to atmospheric MNPs and their toxicity. Respiration is also another important but neglected metabolic pathway through which MNPs can enter the human or animal body. Atmospheric MNPs pose a potential risk as humans breathe 10–20 times per minute on average [84]. MNPs may easily enter the human respiratory system, with people inhaling up to 272 MPs daily indoors [85]. Airborne MNPs exhibit similar aerodynamic characteristics to PM_2.5_ and may reach the deep lung or alveoli upon inhalation. The transport of MNPs within the lung depends on the particles’ size [86]. MNPs are assimilated by human tissues via phagocytic and cell adsorption processes within the respiratory and gastrointestinal systems, precipitating a series of biological responses such as inflammation, cell necrosis, and tissue tearing, which culminate in physical damage. Moreover, MNPs may generate reactive oxygen species, causing chemical damage and a myriad of diseases, including bronchitis, asthma, pneumonia, emphysema, chronic obstructive pneumonia, pulmonary nodules, and lung cancer. However, NPs smaller than 1 μm can pass through lung epithelial cells, crossing the blood–brain barrier and reaching organs such as the placenta, brain, heart, liver, and pancreas and the nervous system [87]. Limited studies exist on the health effects of airborne MNPs, making it necessary to conduct additional research to clarify the relationship between airborne MNPs and human health effects, ultimately enabling the determination of clinical interventions, risk assessments, and the development of public health guidelines.

## 5. Conclusions

The biological ramifications and health risks associated with MNPs have attracted significant attention. By employing bibliometric methods and the WOSCC database, this study analyzed papers published on MNPs’ toxicity from 2014 to 2023. A total of 3541 research articles on MNPs’ toxicity were published, with 77.04% of these papers published within the past three years. The number of articles in this research area exhibited a consistent upward trajectory. The articles predominantly addressed the environmental sciences, engineering, environmental, and toxicology fields. Leading the pack in terms of publication volume was China, followed by Italy and South Korea. Chinese research institutions, such as the Chinese Academy of Sciences, the University of the Chinese Academy of Sciences, and Nanjing University, have published the largest numbers of papers in this field. The top three journals were *Science of The Total Environment*, *Environmental Pollution*, and the *Journal of Hazardous Materials*.

Keyword co-occurrence and burst analyses revealed that the primary topics in the research on MNPs’ toxicity included the influence of MNPs on aquatic organisms, the combined toxicity of MNPs and other contaminants, the toxic effects of MNPs, and the toxicity mechanisms of MNPs. These subject areas are expected to maintain their prominence in future research. Furthermore, future investigations could use computational toxicology and toxicomics techniques to enhance environmental risk assessments and the research on the toxicity mechanisms of MNPs. Expanding the research on the effects of atmospheric MNPs on human health should also be emphasized.

## Figures and Tables

**Table 1:** The top 10 countries/regions in terms of the number of articles published on MNPs’ toxicity from 2014 to 2023.

Countries and Regions	Records	% of 3541	SCPs	MCPs	MCP_Ratio	Total Cited Citations	Average Cited Citations
China	1570	44.34	1297	273	17.39	59,590	37.96
Italy	195	5.51	134	61	31.28	10,946	56.13
South Korea	168	4.74	137	31	18.45	6395	38.07
India	156	4.41	106	50	32.05	3550	22.76
USA	133	3.76	85	48	36.09	8022	60.32
Germany	125	3.53	85	40	32.00	7248	57.98
Spain	117	3.30	78	39	33.33	4594	39.26
Portugal	96	2.71	53	43	44.79	7308	76.13
Brazil	90	2.54	62	28	31.11	2593	28.81
France	71	2.01	46	25	35.21	3303	46.52

Note: Single-Country Publications (SCPs); Multiple-Country Publications (MCPs).

**Table 2:** The top 10 institutions in terms of the number of publications generated that relate to MNPs’ toxicity between 2014 and 2023.

Institution	Country	Records	% of 3541	Total Cited Citations	Average Cited Citations
Chinese Academy of Sciences	China	203	5.73	7595	37.41
University of Chinese Academy of Sciences	China	103	2.91	4127	40.07
Nanjing University	China	76	2.15	7342	96.61
East China Normal University	China	71	2.01	5428	76.45
Southeast University	China	67	1.89	2143	31.99
University of Aveiro	Portugal	65	1.84	3330	51.23
South China Agricultural University	China	57	1.61	1543	27.07
Nankai University	China	56	1.58	1890	33.75
Ocean University of China	China	56	1.58	2854	50.96
Shandong University	China	54	1.52	2575	47.69

**Table 3:** Top 10 authors publishing studies on MNPs’ toxicity from 2014 to 2023.

Author	Country	Records	Total Cited Citations	Average Cited Citations
Wang J	Harbin Institute of Technology (China)	79	2398	30.35
Wang DY	Southeast University (China)	54	1775	32.87
Wang Y	Tibet Agriculture & Animal Husbandry University (China)	50	1619	32.38
Zhang Y	Nanjing University (China)	49	4317	88.10
Wang L	Henan University (China)	43	941	21.88
Liu Y	Zhejiang University of Technology (China)	39	979	25.10
Li J	Nanjing Medical University (China)	33	801	24.27
Lee JS	Sungkyunkwan University (South Korea)	32	2069	64.66
Li Y	Beijing Normal University (China)	32	1084	33.88
Malafaia G	Federal University of Goiás (Brazil)	32	1096	34.25

**Table 4:** The top 10 journals in terms of the number of articles published on MNPs’ toxicity from 2014 to 2023.

Journal Name	Records	IF_2023_	JCR	Total Cited Citations	Average Cited Citations
*Science of The Total Environment*	515	8.2	Q1	23,433	45.50
*Environmental Pollution*	323	7.6	Q1	21,780	67.43
*Journal of Hazardous Materials*	309	12.2	Q1	14,255	46.13
*Chemosphere*	209	8.1	Q1	9407	45.01
*Ecotoxicology and Environmental Safety*	146	6.2	Q1	4371	29.94
*Marine Pollution Bulletin*	105	5.3	Q1	4696	44.72
*Aquatic Toxicology*	93	4.1	Q1	4738	50.95
*Environmental Science & Technology*	87	10.8	Q1	10,428	119.86
*Environmental Science-Nano*	63	5.8	Q1	1975	31.35
*Environment International*	58	10.3	Q1	2830	48.79

**Table 5:** The top 10 most cited publications (articles) related to MNPs’ toxicity published from 2014 to 2023.

Title	Authors	Year	Journal	Total Cited Citations
Uptake and accumulation of polystyrene microplastics in zebrafish (*Danio rerio*) and toxic effects in liver	Lu Y	2016	*Environmental Science & Technology*	1210
Pollutants bioavailability and toxicological risk from microplastics to marine mussels	Avio C G	2015	*Environmental Pollution*	854
Microplastic particles cause intestinal damage and other adverse effects in zebrafish *Danio rerio* and nematode Caenorhabditis elegans	Lei L	2018	*Science of the Total Environment*	788
Microplastics in seafood and the implications for human health	Smith M	2018	*Current Environmental Health Reports*	776
Microplastic size-dependent toxicity, oxidative stress induction, and p-JNK and p-p38 activation in the monogonont rotifer (*Brachionus koreanus*)	Jeong C B	2016	*Environmental Science & Technology*	758
Tissue accumulation of microplastics in mice and biomarker responses suggest widespread health risks of exposure	Deng Y	2017	*Scientific Reports*	666
Differentially charged nanoplastics demonstrate distinct accumulation in *Arabidopsis thaliana*	Sun X D	2020	*Nature Nanotechnology*	532
Polystyrene microplastics induce gut microbiota dysbiosis and hepatic lipid metabolism disorder in mice	Lu L	2018	*Science of the Total Environment*	492
Impacts of polystyrene microplastic on the gut barrier, microbiota and metabolism of mice	Jin Y X	2019	*Science of the Total Environment*	479
Toxic effects of microplastic on marine microalgae *Skeletonema costatum*: Interactions between microplastic and algae	Zhang C	2017	*Environmental Pollution*	476

**Table 6:** The top 10 most cited publications (reviews) related to MNPs’ toxicity published from 2014 to 2023.

Title	Authors	Year	Journal	Total Cited Citations
Environmental exposure to microplastics: An overview on possible human health effects	Prata J C	2020	*Science of the Total Environment*	904
Airborne microplastics: Consequences to human health?	Prata J C	2018	*Environmental Pollution*	700
Potential health impact of environmentally released micro-and nanoplastics in the human food production chain: experiences from nanotoxicology	Bouwmeester H	2015	*Environmental Science & Technology*	687
Source, migration and toxicology of microplastics in soil	Guo J J	2020	*Environment International*	488
Ecotoxicological effects of microplastics on biota: a review	Anbumani S	2018	*Environmental Science and Pollution Research*	423
A meta-analysis of the effects of exposure to microplastics on fish and aquatic invertebrates	Foley C J	2018	*Science of the Total Environment*	386
Recent advances in toxicological research of nanoplastics in the environment: A review	Shen M	2019	*Environmental Pollution*	351
Potential human health risks due to environmental exposure to nano-and microplastics and knowledge gaps: A scoping review	Rahman, A	2021	*Science of the Total Environment*	310
Ecotoxicological effects of microplastics: Examination of biomarkers, current state and future perspectives	Prokić M D	2019	*TrAC Trends in Analytical Chemistry*	299
Micro-(nano) plastics in freshwater ecosystems: abundance, toxicological impact and quantification methodology	Strungaru S A	2019	*TrAC Trends in Analytical Chemistry*	288

**Table 7:** The main keywords in the four clusters.

Cluster	Research Topic	Main Keywords
Cluster I	toxicity to marine organisms	marine environment (354); ingestion (504); acute toxicity (82); daphnia magna (52); survival (42); zooplankton (75)
Cluster II	joint toxicity	bioavailability (68); polycyclic aromatic hydrocarbons (79); additives (91); heavy metals (81); bisphenol a (104); flame retardant (52); chemicals (109)
Cluster III	toxic effect	growth (299); accumulation (444); impact (301); health (106); behavior (144); uptake (38); size (151)
Cluster IV	mechanism of toxicity	exposure (692); oxidative stress (705); bioaccumulation (233); biomarkers (137); gene expression (117); responses (173); metabolism (140); toxicology (96); inflammation (126); DNA damage (20)

## Data Availability

The raw data supporting the conclusions of this article will be made available by the authors on request.
